# Supplementary material for: SEN virus genotype H distribution in β-thalassemic patients and in healthy donors in Iraq: Molecular and physiological study
Source: PLoS Negl Trop Dis. 2020 Jun 8;14(6):e0007880. doi: 10.1371/journal.pntd.0007880 (PMC7302744; doi:10.1371/journal.pntd.0007880)
Supplement: S1 Checklist — (DOC) [file pntd.0007880.s001.doc]

S1 STROBE Statement—Checklist of items that should be included in reports of ***cross-sectional studies***

|  | Item No | Recommendation |
| --- | --- | --- |
| **Title and abstract** | 1 | (*a*) Indicate the study’s design with a commonly used term in the title or the abstract  The study type is not specifically mentioned in the title but the type of study is implied. |
|  |  | (*b*) Provide in the abstract an informative and balanced summary of what was done and what was found  The abstract is on p. 2 |
| Introduction | | |
| Background/rationale | 2 | Explain the scientific background and rationale for the investigation being reported  The Introduction (pp 3-4) comprises the background |
| Objectives | 3 | State specific objectives, including any prespecified hypotheses  Found in the last paragraph of the Introduction |
| Methods | | |
| Study design | 4 | Present key elements of study design early in the paper  The key elements of the study are found in the Methods section |
| Setting | 5 | Describe the setting, locations, and relevant dates, including periods of recruitment, exposure, follow-up, and data collection  Methods – Paragraph 1 |
| Participants | 6 | (*a*) Give the eligibility criteria, and the sources and methods of selection of participants  Methods – Paragraph 1 |
| Variables | 7 | Clearly define all outcomes, exposures, predictors, potential confounders, and effect modifiers. Give diagnostic criteria, if applicable  Generally not applicable, but some diagnostic criteria in Methods – Paragraph 1 |
| Data sources/ measurement | 8* | For each variable of interest, give sources of data and details of methods of assessment (measurement). Describe comparability of assessment methods if there is more than one group  Described in detail in appropriate sections of the Methods |
| Bias | 9 | Describe any efforts to address potential sources of bias  The nature of the study prevented specific controls for bias (noted as a limitation of the study, 2^nd^ last paragraph of the Discussion. |
| Study size | 10 | Explain how the study size was arrived at  Covered in Methods – Paragraph 1 |
| Quantitative variables | 11 | Explain how quantitative variables were handled in the analyses. If applicable, describe which groupings were chosen and why  Covered in the Statistical Analysis section of the Methods |
| Statistical methods | 12 | (*a*) Describe all statistical methods, including those used to control for confounding |
|  |  | (*b*) Describe any methods used to examine subgroups and interactions |
|  |  | (*c*) Explain how missing data were addressed |
|  |  | (*d*) If applicable, describe analytical methods taking account of sampling strategy |
|  |  | (*e*) Describe any sensitivity analyses  The relevant statistical manipulations are described in the Statistical Analysis section of the Methods |
| Results | | |
| Participants | 13* | (a) Report numbers of individuals at each stage of study—eg numbers potentially eligible, examined for eligibility, confirmed eligible, included in the study, completing follow-up, and analysed |
|  |  | (b) Give reasons for non-participation at each stage |
|  |  | (c) Consider use of a flow diagram  The study involved all-comers meeting the requirements outlined in the Methods section – reported in Paragraph 1 of the Results. |
| Descriptive data | 14* | (a) Give characteristics of study participants (eg demographic, clinical, social) and information on exposures and potential confounders  In the interest of the length of the paper not all assessed characteristics are reported. Key characteristics are described in the Tables. |
|  |  | (b) Indicate number of participants with missing data for each variable of interest  Not applicable. |
| Outcome data | 15* | Report numbers of outcome events or summary measures  Presented in tables. |
| Main results | 16 | (*a*) Give unadjusted estimates and, if applicable, confounder-adjusted estimates and their precision (eg, 95% confidence interval). Make clear which confounders were adjusted for and why they were included |
|  |  | (*b*) Report category boundaries when continuous variables were categorized |
|  |  | (*c*) If relevant, consider translating estimates of relative risk into absolute risk for a meaningful time period  Presented, as necessary, in the tables. |
| Other analyses | 17 | Report other analyses done—eg analyses of subgroups and interactions, and sensitivity analyses  Not applicable |
| Discussion | | |
| Key results | 18 | Summarise key results with reference to study objectives  Discussion – paragraph 1 |
| Limitations | 19 | Discuss limitations of the study, taking into account sources of potential bias or imprecision. Discuss both direction and magnitude of any potential bias  Discussion – second last paragraph |
| Interpretation | 20 | Give a cautious overall interpretation of results considering objectives, limitations, multiplicity of analyses, results from similar studies, and other relevant evidence  Discussion – last paragraph |
| Generalisability | 21 | Discuss the generalisability (external validity) of the study results  Discussion – second last paragraph |
| Other information | | |
| Funding | 22 | Give the source of funding and the role of the funders for the present study and, if applicable, for the original study on which the present article is based  None, as stated after the discussion |

*Give information separately for exposed and unexposed groups.

**Note:** An Explanation and Elaboration article discusses each checklist item and gives methodological background and published examples of transparent reporting. The STROBE checklist is best used in conjunction with this article (freely available on the Web sites of PLoS Medicine at http://www.plosmedicine.org/, Annals of Internal Medicine at http://www.annals.org/, and Epidemiology at http://www.epidem.com/). Information on the STROBE Initiative is available at www.strobe-statement.org.
